# Symptomatic Atherosclerotic Non-acute Intracranial Vertebral Artery Total Occlusion: Clinical Features, Imaging Characteristics, Endovascular Recanalization, and Follow-up Outcomes

**DOI:** 10.3389/fneur.2020.598795

**Published:** 2020-11-16

**Authors:** Wei Zhao, Jun Zhang, Yao Meng, Yuyan Zhang, Jinping Zhang, Yun Song, Lili Sun, Meimei Zheng, Wei Wang, Hao Yin, Ju Han

**Affiliations:** Department of Neurology, The First Affiliated Hospital of Shandong First Medical University and Shandong Provincial Qianfoshan Hospital, Jinan, China

**Keywords:** atherosclerosis, non-acute intracranial vertebral artery occlusion, features, endovascular treatment, outcome

## Abstract

**Background and Objectives:** Previous studies on symptomatic atherosclerotic non-acute intracranial vertebral artery total occlusion that was refractory to medical therapy are rare. We aimed to assess the clinical features, imaging characteristics, endovascular treatment feasibility and follow-up outcomes of patients with this condition.

**Methods:** Data from consecutive patients who had symptomatic atherosclerotic non-acute intracranial vertebral artery total occlusion and underwent endovascular recanalization from February 2016 to April 2020 were retrospectively collected in our prospective database. Clinical, imaging, procedural, and follow-up data were collected and analyzed.

**Results:** Thirty-one patients, predominantly males, were enrolled in this study. These patients presented with recurrent/progressive stroke in the posterior circulation despite aggressive medical therapy. Angiographic analysis revealed asymmetric vertebral arteries due to unilateral hypoplasia and intracranial vertebral artery total occlusions in the dominant vertebral arteries, which were characterized by long lesion length and high clot burden. Multiple infarctions and perfusion defects in the posterior circulation were demonstrated by diffusion-weighted imaging and arterial spin labeling, respectively. Successful endovascular recanalization was achieved in 87.1% of the patients. Over a median clinical follow-up duration of 11.0 months, 74.1% of patients with successful recanalization achieved favorable clinical outcomes (mRS score ≤2).

**Conclusion:** Symptomatic atherosclerotic non-acute intracranial vertebral artery total occlusion attributable to hypoperfusion is characterized by recurrent/progressive ischemic events, dominant intracranial vertebral artery total occlusion, long lesion length, and high clot burden. Endovascular recanalization of the dominant intracranial vertebral artery occlusion appears to be a feasible treatment for these patients.

## Introduction

The intracranial vertebral artery (ICVA) is a common site of atherosclerotic occlusion that is often involved bilaterally; however, the ICVA has received the least attention, especially concerning the treatment of occlusive lesions (1). A subset of patients survive the acute ICVA occlusion stage and continue to suffer recurrent strokes and transient ischemic attacks (TIAs) in posterior circulation despite aggressive medical therapy in the subacute and chronic period (1–3). Hypoperfusion without adequate collateral circulation is a main mechanism for recurrent ischemic strokes and worsening symptoms, and it is highly likely that medical therapy will fail in these patients; rather, they will benefit from revascularization (4, 5).

Previous studies have been rare and are limited by small sample sizes of patients with symptomatic atherosclerotic non-acute ICVA total occlusion that was refractory to medical therapy (6–12). We aimed to assess the clinical features, imaging characteristics, endovascular treatment feasibility, and follow-up outcomes of these patients.

## Materials and Methods

### Study Population

“Non-acute occlusion” was defined as symptomatic (TIA or stroke) complete occlusion of an intracranial artery of presumed atherosclerotic etiology in which endovascular therapy was performed more than 48 h from the time the patients was last seen well (6). We retrospectively reviewed our prospective stroke intervention database to identify consecutive patients who had symptomatic atherosclerotic non-acute ICVA total occlusion and underwent endovascular recanalization from February 2016 to April 2020. The Institutional Review Board of the First Affiliated Hospital of Shandong First Medical University approved the study.

The inclusion criteria were as follows: (1) intracranial atherosclerosis was the primary etiology; (2) experienced recurrent TIAs or stroke (neurological deterioration, such as deterioration of consciousness, hemiparesis, sensory disturbance, ataxia, dizziness, vertigo, dysarthria, dysphagia, diplopia, etc.) related to occluded vertebral artery despite aggressive medical treatment, which was defined as the treatment including dual-antiplatelet therapy, statin, blood pressure and glucose control, smoking cessation and an emphasis on healthy lifestyle; (3) total occlusion of dominant ICVA was confirmed by DSA; and (4) hemodynamic failure and hypoperfusion in the ICVA territory were confirmed based on the clinical and imaging evidence.

The exclusion criteria were as follows: (1) non-atherosclerotic occlusion, such as vasculitis, arterial dissection, or embolic disease; (2) clinical symptoms were stable with aggressive medical treatment; (3) contraindications to operation, such as known allergy or contraindication to aspirin, clopidogrel, or anesthesia; and (4) life expectancy <1 year because of other medical conditions.

### Clinical Assessment

We assessed the patient demographic information and cardiovascular risk factors, including age, sex, hypertension, diabetes mellitus, hyperlipidemia, previous history of stroke, coronary artery disease, atrial fibrillation, and smoking. The modified Rankin scale (mRS) scores and the National Institutes of Health Stroke Scale (NIHSS) scores were determined by well-trained neurologists.

### Radiological Assessment

ICVA total occlusion was initially assessed by non-invasive computed tomography angiography (CTA) or magnetic resonance angiography (MRA) and then confirmed by digital subtraction angiography (DSA). Total occlusion was defined as grade 0 antegrade flow distal to the occlusion by thrombolysis in the cerebral infarction (TICI) grading system on DSA. High-resolution magnetic resonance imaging (HRMRI) was used to analyze the occlusion etiology, occlusion course and luminal thrombosis of these patients. Magnetic resonance imaging (MRI) and arterial spin labeling (ASL) were used to assess multiple infarctions and regional hypoperfusion defects in the ICVA territory, respectively. Posterior circulation acute stroke prognosis early CT scores (pc-ASPECTS) on diffusion-weighted imaging (DWI), which were determined within 3 days before the procedure, were provided by well-trained neurologists.

### Intervention Procedure

Dual antiplatelet treatment with 100 mg aspirin and 75 mg clopidogrel daily was routinely maintained for at least 5 days before the procedure, and thromboelastography platelet mapping was performed to guide the modulation of antiplatelet treatment. The details of the interventional procedure have been described previously (13, 14). The lesions were initially predilated with conventional balloons (Gateway balloon, Boston Scientific, USA). Drug-coated balloon (DCB) (SeQuent Please, B. Braun, Germany) have been applied after conventional balloon angioplasty to inhibit intimal hyperplasia and restenosis in later research (15, 16). When the residual stenosis was >50% and the antegrade perfusion was unstable, or there was vessel dissection after balloon angioplasty, remedial stenting implantation was performed (Wingspan stent, Stryker Neurovascular, USA; Solitaire AB stent, Medtronic, United States; Apollo stent, Micro-port Neuro Tech, China, Xience Prime stent, Abbott Vascular, United States) (17). Transcatheter aspiration was applied to reduce the clot burden prior to angioplasty when the clot burden was high proximal to the occlusion segment. Intravenous low dose tirofiban injection was administered when there were obvious clots at and around the occlusion lesions.

Post-procedural antegrade flow was graded using the TICI grading system, and technical success was determined by recanalization with a TICI grade ≥2b on post-procedural angiography. Procedure complications included perforating branch occlusion, embolization, hyperperfusion syndrome, intracranial hemorrhage (ICH), subarachnoid hemorrhage, vessel perforation and dissection.

### Follow-up Outcomes

All patients were discharged on dual antiplatelets, consisting of aspirin and clopidogrel, and were required to remain on the dual antiplatelet regimen for 3 months for angioplasty and 6 months for stenting, after which they continued with one of the two drugs. Patients were also treated with statins and other risk factor controls.

These patients were followed up clinically at 1 month, and all the patients were followed up clinically in May 2020. They were scheduled for DSA at 3–6 months. Favorable functional outcome was defined as an mRS score 0–2. Restenosis was defined as a diameter of the stenosis >50% of the target artery segment. Symptomatic restenosis was defined as restenosis associated with ischemic symptoms of the treated vessel territory.

### Statistical Analysis

Continuous data are expressed as the mean ± standard deviation (SD) or as the median with the interquartile range (IQR). Categorical data are expressed as numbers and percentages. Statistical analysis was performed using SPSS version 19.0 for Windows (SPSS Inc., Chicago, IL, United States).

## Results

### Clinical Features

Thirty-one patients, predominantly males (27/31, 87.1%), were enrolled in this study (Supplementary Table 1). Baseline clinical characteristics are listed in Table 1. The mean age of the patients was 58.9 ± 8.5 years. The most common risk factors were hypertension (29/31, 93.5%), smoking (22/31, 71.0%), diabetes mellitus (14/31, 45.2%), coronary artery disease (11/31, 35.5%) and previous history of stroke (8/31, 25.8%).

**Table 1:** Baseline clinical variables.

**Baseline clinical variables**	***N* = 31 (%)**
Age(years), mean (SD)	58.9 ± 8.5
Male	27 (87.1)
Hypertension	29 (93.5)
Diabetes mellitus	14 (45.2)
Coronary artery disease	11 (35.5)
Previous history of stroke	8 (25.8)
Hyperlipidemia	2 (6.5)
Atrial fibrillation	2 (6.5)
Smoking	22 (71.0)
Pre-treatment NIHSS, median (IQR)	4 (2–7)
Pre-treatment mRS, median (IQR)	3 (2–4)
Pre-treatment pc-ASPECTS on DWI, median (IQR)	6 (5–7)

*NIHSS, National Institutes of Health Stroke Scale; mRS, modified Rankin scale; pc-ASPECTS, posterior circulation acute stroke prognosis early CT score; DWI, diffusion-weighted imaging; SD, standard deviation; IQR, interquartile range*.

All these patients were treated with aggressive medical therapy since presentation and still experienced recurrent and progressive strokes in the posterior circulation. Common symptoms included dizziness and vertigo, dysarthria, dysphagia, diplopia, blurred vision, ataxia, hemiparesis, sensory disturbance, headache, hearing loss, and so on. Lifestyle-limiting symptoms that were exacerbated by activity or decreases in blood pressure, such as aggravated dizziness and vertigo, blurred vision, ataxia, and headache after standing or walking, were prominent in these patients.

The pretreatment median mRS and NIHSS scores at baseline were 3 (IQR, 2–4) and 4 (IQR, 2–7), respectively. A total of 61.3% (19/31) of these patients had low NIHSS scores (<6), whereas these patients also had poor quality of life because of serious vertigo, dysphagia, diplopia, or ataxia.

### Imaging Characteristics

**Figure 1:**
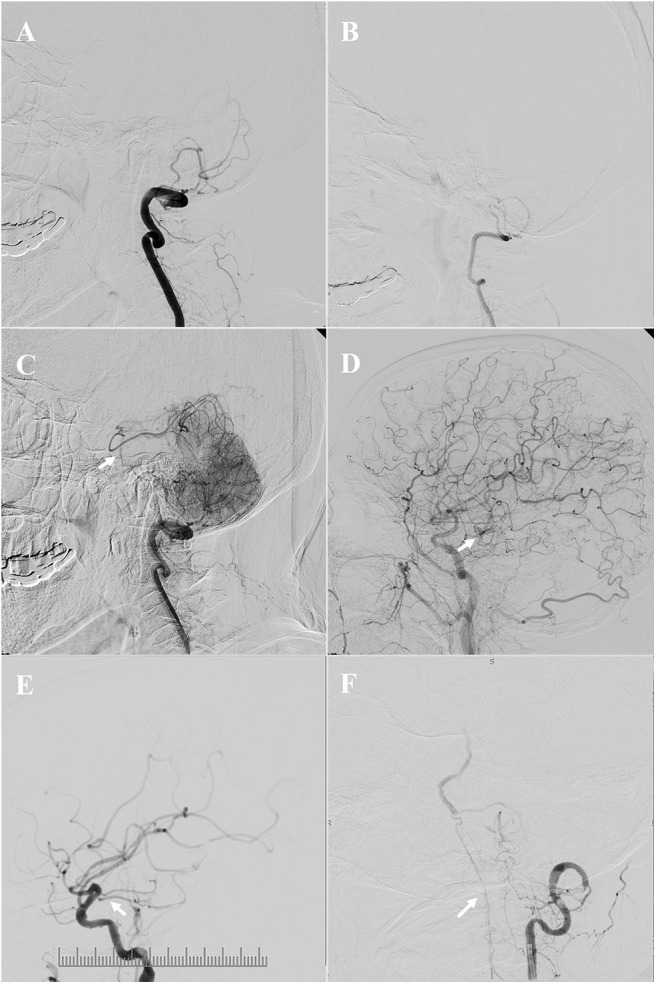
Angiographic features and collateral circulation. Illustrative patient 18 **(A**–**D)**: asymmetric vertebral arteries (VA) due to unilateral hypoplasia, the dominant intracranial vertebral artery (ICVA) was totally occluded **(A)**, the blood flow of the contralateral hypoplastic VA was tenuous, and ended in the posterior inferior cerebellar artery (PICA) **(B)**; the collateral flow from posterior and anterior leptomeningeal anastomosis was limited at late arterial phases [**(C,D)**, the arrow indicates the top of the basilar artery]. Illustrative patient 1**(E)**: the arrow indicates a tiny posterior communicating artery (PComA). Illustrative patient 19 **(F)**: the arrow indicates upward retrograde flow through the anterior spinal artery (ASA).

Angiographic analysis revealed that the vertebral arteries (VA) of all these patients were asymmetric due to unilateral hypoplasia, and ICVA total occlusions occurred in the dominant VA (Figure 1). The blood flow of the contralateral hypoplastic VA was tenuous, as 71.0% (22/31) of the contralateral hypoplastic VA ended in the posterior inferior cerebellar artery (PICA), 9.7% (3/31) of the contralateral hypoplastic VA ended before entering the skull, 6.5% (2/31) of the contralateral hypoplastic VA had multiple serious stenoses, and 12.9% (4/31) of the contralateral hypoplastic VA had whole-course extreme hypoplasia.

MRI revealed multiple infarcts in the cerebellum, medulla oblongata, pontine, midbrain, thalamus, splenium of the corpus callosum, and temporal and occipital lobes in the ICVA territory. Pre-treatment pc-ASPECTS on DWI was 6 (IQR, 5–7).

**Figure 2:**
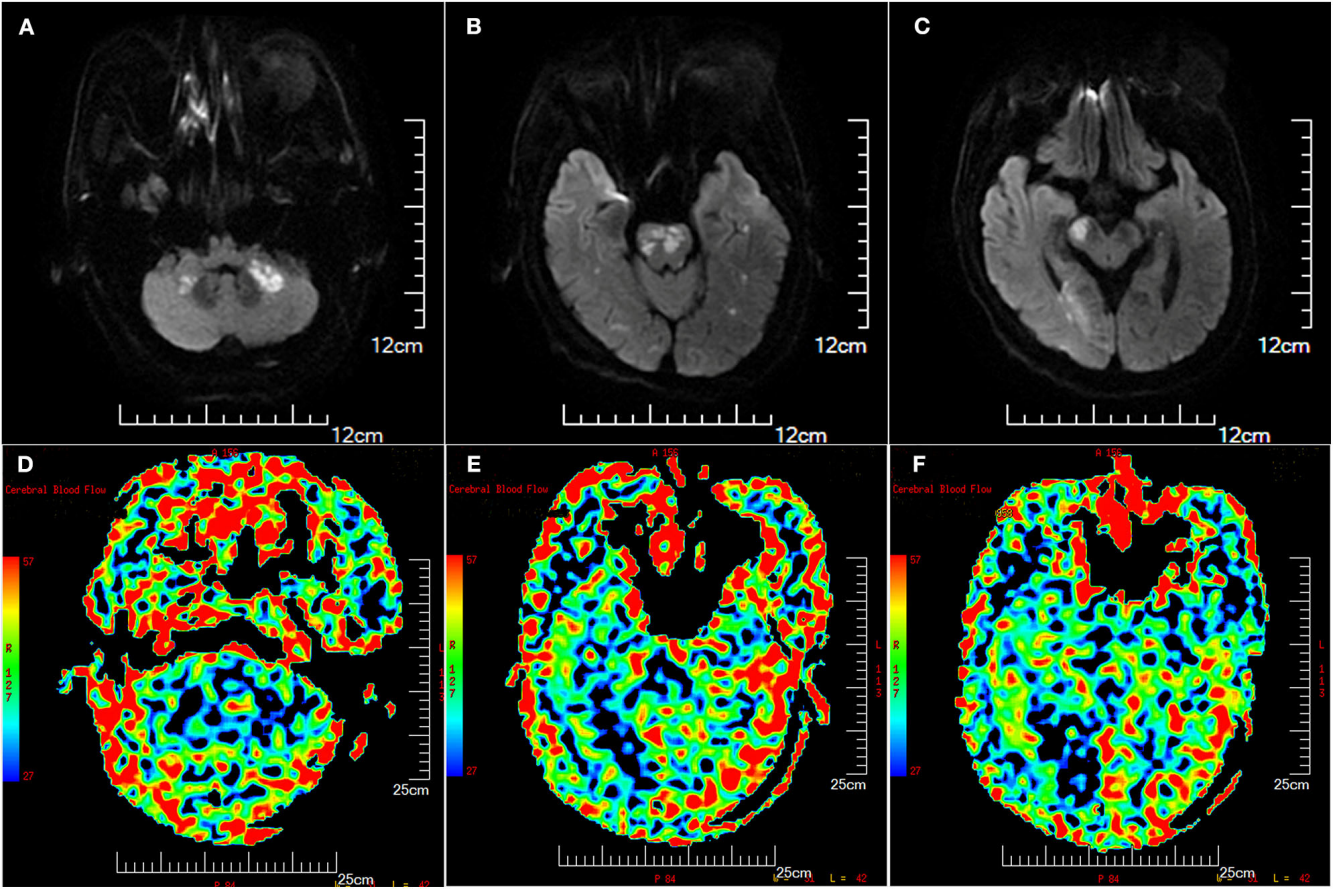
Illustration of multiple infarctions and perfusion defects in the posterior circulation (patient 8), which were detected by diffusion-weighted imaging (DWI) **(A–C)** and arterial spin labeling (ASL) **(D–F)**, respectively. The Scale for ASL [image **(D–F)**] was color coded (red, largest cerebral blood flow; blue, least cerebral blood flow). ASL images showed larger perfusion deficits including the brain stem, cerebellum, and occipital lobe.

A total of 25.8% (8/31) of the patients had a posterior communicating artery (PComA), 38.7% (12/31) of the patients had upward retrograde flow through the anterior spinal artery (ASA), and 6.5% (2/31) of the patients had PComA and upward retrograde flow through the ASA. However, the PComA was tiny in these patients, and the collateral blood flow of these patients through PComA, ASA, and leptomeningeal anastomosis was tenuous and limited (Figure 1). The American Society of Interventional and Therapeutic Neuroradiology/Society of Interventional Radiology Collateral Flow Grading System score of all these patients was <3 on DSA. Relatively small and multiple infarctions on DWI with a large area of low perfusion assessed by ASL demonstrated hypoperfusion defects in the ICVA territory of these patients (Figure 2).

**Figure 3:**
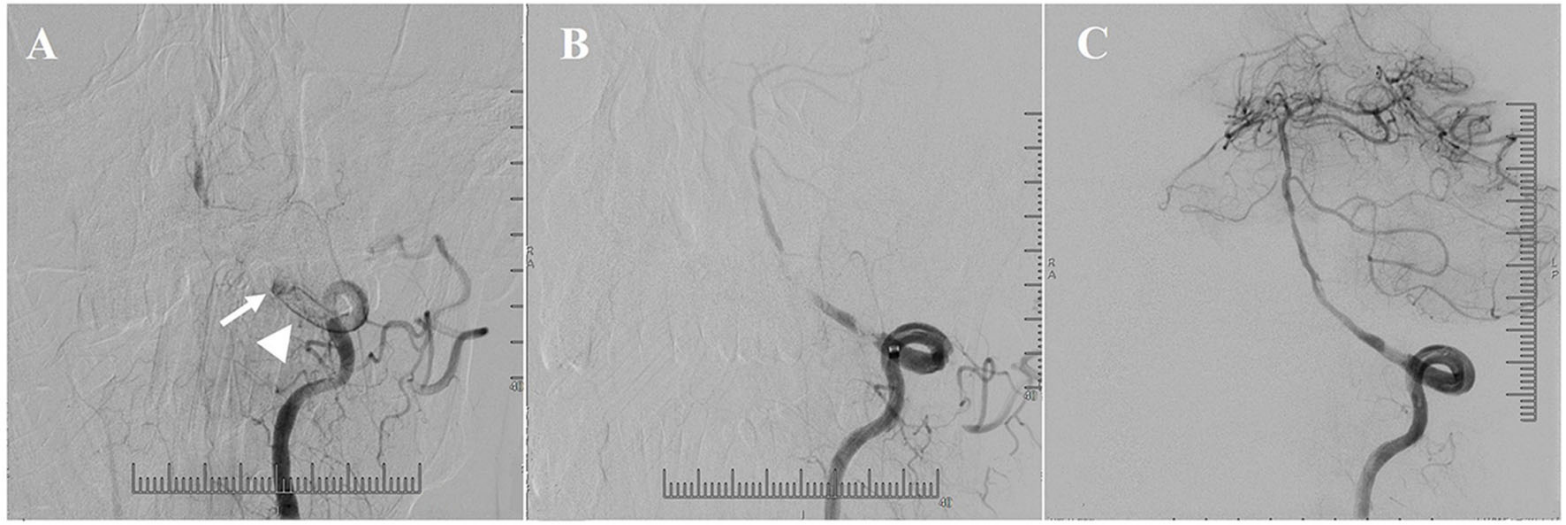
Illustrative patient 20. **(A)** A lot of clots (arrowhead) proximal to the occlusion segment (arrow). **(B)** Angiographic result after transcatheter aspiration. **(C)** Favorable antegrade flow was obtained after conventional balloon and drug-coated balloon (DCB) angioplasty.

**Figure 4:**
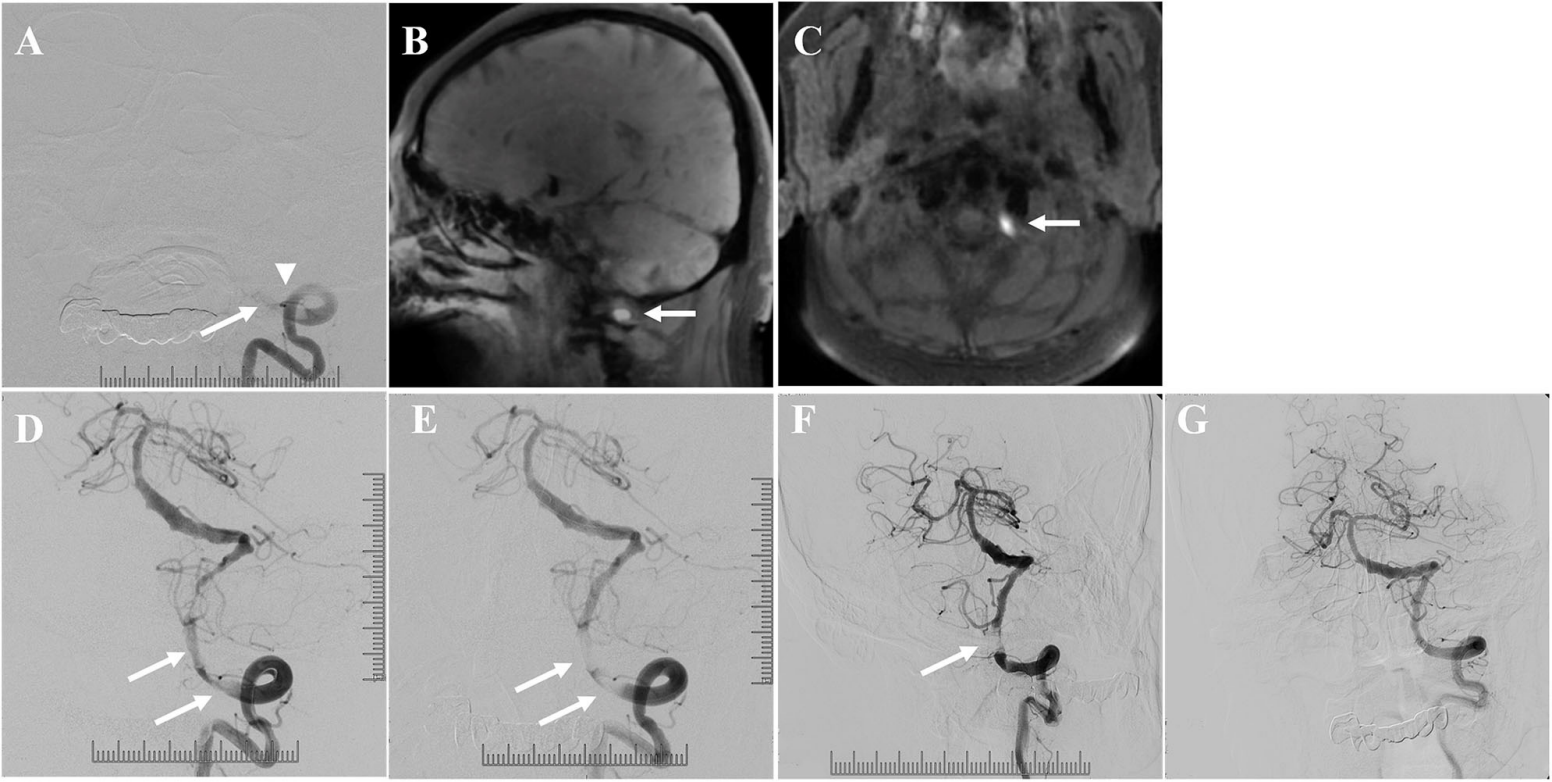
Illustrative patient 14: **(A)** Total occlusion (arrow) of the ICVA with obvious clots at and proximal to the occlusion segment (arrowhead) on digital subtraction angiography (DSA). **(B,C)** Clots with high signal intensity (arrow) on pre-contrast T1-weighted high-resolution magnetic resonance imaging (HRMRI). **(D)** Angiographic result after conventional balloon angioplasty. There were obvious clots (arrow). **(E)** Angiographic results after stenting demonstrated favorable antegrade flow, despite persistent clots (arrow). **(F)** The patient was treated with intravenous low-dose tirofiban injection and dual antiplatelets for 7 days. DSA 7 days later showed good antegrade flow with interval reduction in clot burden (the arrow indicates the residual clots). **(G)** Then, the patient was treated with dual antiplatelets after discharge. DSA 5 months later showed that the antegrade flow was good, without restenosis or obvious clots.

**Figure 5:**
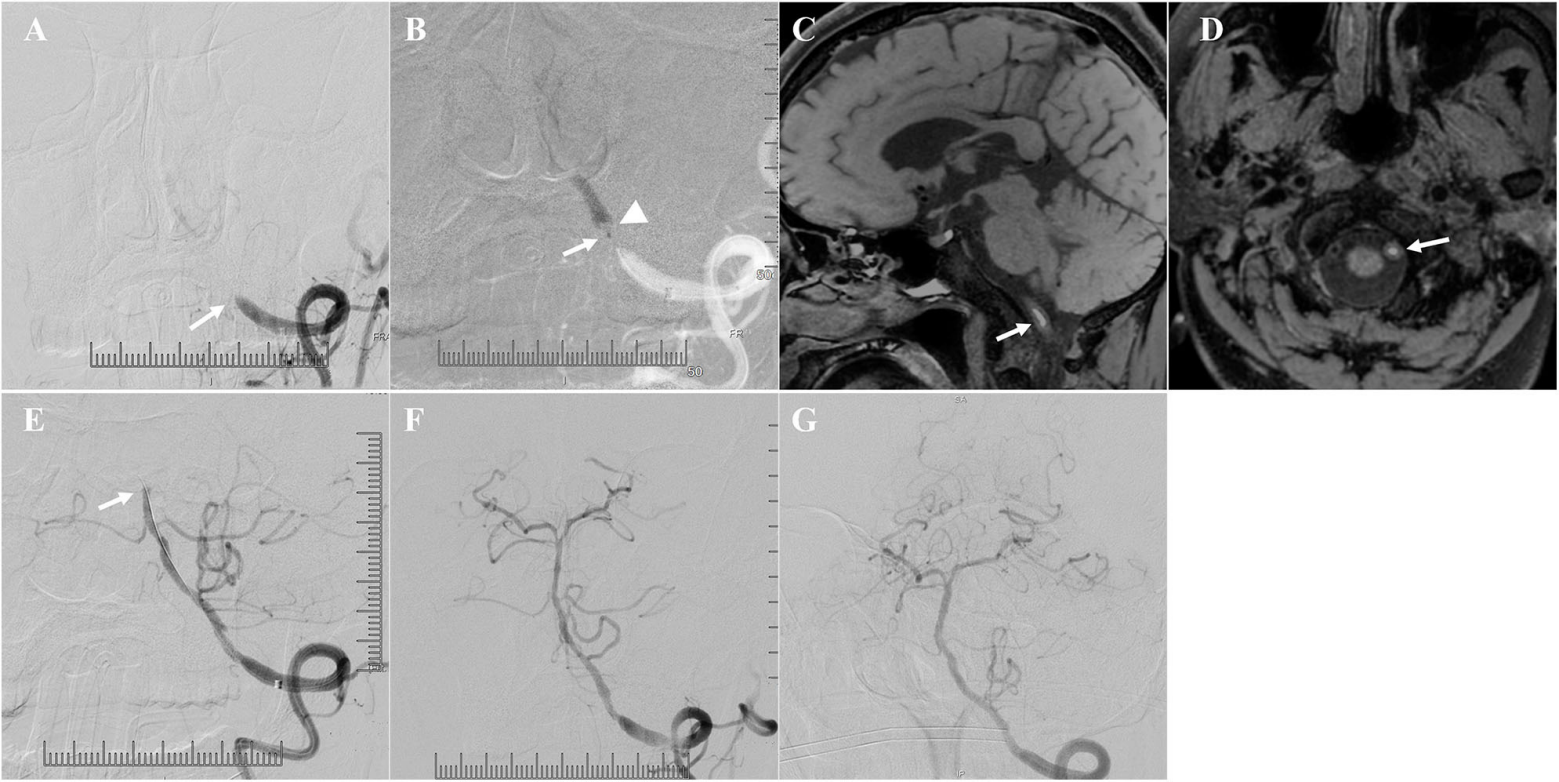
Illustrative patient 30. **(A)** Total occlusion of the intracranial vertebral artery (ICVA) (arrow). **(B)** Microcatheter angiography (the arrow indicates the position of the microcatheter) showed a relatively short occlusion segment and obvious filling defects distal to the occlusion segment (arrowhead). The filling defects were clots distal to the occlusion segment. **(C,D)** Clots with high signal intensity distal to the occlusion segment on pre-contrast T1-weighted high-resolution magnetic resonance imaging (HRMRI) (arrow). **(E)** Angiographic results after conventional balloon and drug-coated balloon angioplasty demonstrated migration of the clots to the distal segment of the basilar artery. Distal antegrade flow could not be seen (arrow). **(F)** Favorable antegrade flow was obtained after emergency transcatheter aspiration. **(G)** Angiography 3 months later demonstrated favorable antegrade flow without restenosis.

Obvious clots occurred in 41.9% (13/31) of the patients at or around the ICVA occlusion lesions, which was detected by DSA and HRMRI. The clots were divided into three categories according to the location: occlusion segment, distal to the occlusion segment, and proximal to the occlusion segment (Figures 3–5).

### Endovascular Treatment

The endovascular procedure was applied for the dominant ICVA total occlusion of these patients. The median time from symptom onset to endovascular treatment was 23.0 days (IQR, 15.0–53.5 days), and the median time from image-documented ICVA total occlusion to endovascular treatment was 14.0 days (IQR, 7.0–29.5 days).

Transcatheter aspiration prior to angioplasty was able to effectively reduce the clot burden proximal to the occlusion segment (Figure 3). Favorable antegrade flow was achieved after angioplasty and stenting despite obvious clots at and proximal to the occlusion segment (Figures 3, 4).

Successful recanalization was achieved in 87.1% (27/31) of the patients, with TICI 3 reperfusion in 25 cases (80.6%, 25/31) and TICI 2b reperfusion in 2 cases (6.5%, 2/31). The treatment modalities and outcomes of the patients are summarized in Table 2. DCBs were used in 15 cases after conventional balloon dilatation to inhibit intimal hyperplasia. A total of 44.4% (12/27) of the patients had a long lesion length and underwent stenting with a stent ≥20 mm in length. One patient was treated with two Wingspan stents (4 ^*^ 15 mm, 4.5 ^*^ 20 mm) because of the long lesion length, and the other 11 patients were treated with one stent that was ≥20 mm in length (one patient was treated with a Xience Prime stent that was 28 mm in length, three patients were treated with a Xience Prime stent that was 23 mm in length, six patients were treated with a Wingspan stent that was 20 mm in length, and one patient was treated with a Solitaire AB stent that was 20 mm in length). The median residual stenosis after the procedure was 15.0% (IQR, 10.0–25.0%).

**Table 2:** Procedural characteristics.

**Variables**	***N* = 31 (%)**
Symptom onset to treatment (days), median (IQR)	23.0 (15.0–53.5)
Image-documented occlusion to treatment (days), median (IQR)	14.0 (7.0–29.5)
Technical success	27 (87.1)
**Modality of recanalization**	
CBA	3 (9.7)
CBA+stenting	9 (29.0)
CBA+DCBA	8 (25.8)
CBA+DCBA+stenting	7 (22.6)
**Post-procedural perfusion**	
TICI 3	25 (80.6)
TICI 2b	2 (6.5)
TICI 0	4 (12.9%)
Residual stenosis, median (IQR)	15.0% (10.0–25.0)
Complication rate	6 (19.4)
Perforating branch occlusion	2 (6.5)
Embolization	2 (6.5)
Dissection	1 (3.2)
Asymptomatic ICH	1 (3.2)
Symptomatic complication rate	3 (9.7)
Perforating branch occlusion	2 (6.5)
Dissection	1 (3.2)

*CBA, conventional balloon angioplasty; DCBA, drug-coated balloon angioplasty; TICI, thrombolysis in cerebral infarction; ICH, intracranial hemorrhage; IQR, interquartile range*.

The procedure failed in four patients because the guidewire could not traverse the occluded segment. Six patients experienced procedural related complications, of whom only three patients were symptomatic. Perforating branch occlusion occurred in two patients, and symptomatic dissection occurred in one patient. The three patients had new infarcts in posterior circulation after the procedure, and their mRS scores were 1, 3, and 3 at the last follow-up, respectively.

Asymptomatic ICH occurred in one patient after the procedure. Hemorrhage was detected in the previous occipital lobe infarction, which may have resulted from hyperperfusion after recanalization. Asymptomatic embolization occurred in two patients who had clots distal to the occlusion lesions, and neither of them exhibited new clinical manifestations with emergency mechanical thrombectomy (MT) (Figure 5). Other periprocedural complications, such as subarachnoid hemorrhage or perforation, did not occur in this case series.

### Clinical and Angiographic Follow-up Data

The clinical and angiographic follow-up outcomes of successfully treated patients are presented in Table 3. The lifestyle-limiting symptoms improved quickly within several days in the patients with successful recanalization. Unexpectedly, the symptom of hearing loss also improved quickly within several days after the procedure. The symptom improvement rate after the procedure was 85.2% (23/27) for these patients. At the first 30-day clinical follow-up, there were no recurrent cerebral ischemic events; 66.7% (18/27) of the patients achieved a favorable clinical outcome (mRS score ≤2), and 85.2% (23/27) of the patients achieved an acceptable clinical outcome (mRS score ≤3). Over a median clinical follow-up duration of 11.0 months, 74.1% (20/27) of the patients achieved a favorable clinical outcome (mRS score ≤2), and 88.9% (24/27) of the patients achieved an acceptable clinical outcome (mRS score ≤3) at the last follow-up. There was one death due to multiple organ failure.

**Table 3:** Clinical and angiographic outcomes of successfully treated patients.

**Variables**	***N* = 27 %**	**Median (IQR)**
Follow-up time(months)		11.0(5.0–26.5)
Symptom improved post-procedure	23 (85.2)	
30-day mRS score ≤2	18 (66.7)	
30-day mRS score ≤3	23 (85.2)	
mRS score at last follow-up ≤2	20 (74.1)	
mRS score at last follow-up ≤3	24 (88.9)	
Ischemic event during follow-up	1 (3.2)	
Death	1 (3.2)	
Restenosis on follow-up image	10% (2/20)	

*mRS, modified Rankin scale; IQR, interquartile range*.

During the 5.5 ± 2.6 months vessel imaging follow-up period, DSA was obtained for 18 patients, and CTA was obtained for 2 patients. Angiographic follow-up demonstrated continuous clot dissolution in these patients after successful endovascular recanalization (Figure 4). Restenosis occurred in 10% (2/20) of patients who had follow-up imaging: one presented with angiographic asymptomatic restenosis; whereas the other presented with symptomatic reocclusion 7.5 months after the procedure, and this patient was neurologically independent with emergency MT and stenting, the mRS of this patient was 0 before discharge. No recurrent stroke occurred in other patients with successful recanalization during the clinical follow-up period.

## Discussion

In our study, patients with symptomatic atherosclerotic non-acute ICVA total occlusion were treated with aggressive medical therapy since presentation, but they were still hemodynamically unstable and experienced recurrent and progressive ischemic events despite aggressive medical therapy. Therefore, they were transferred to our comprehensive stroke center for further treatment. These patients had asymmetric VA due to unilateral hypoplasia, and ICVA total occlusions occurred in the dominant VA. The blood flow of the contralateral hypoplastic VA was tenuous, and most of them ended in the PICA. This condition exposed these patients to the risk of a catastrophic stroke in the basilar artery territory.

Limitations of NIHSS include a focus on limb and speech impairments and less emphasis on cranial nerve lesions. Patients with symptomatic ICVA total occlusions may have low NIHSS scores, whereas the mRS scores of these patients can be high because of serious vertigo, dysphagia, diplopia, or ataxia, so we did not stratify the patients by NIHSS score. Pc-ASPECTS on DWI is helpful in predicting functional outcome in posterior circulation ischemic stroke (18), and the low pretreatment median pc-ASPECTS (6, IQR, 5–7) on DWI indicated a poor prognosis for these patients.

The collateral flow from PComA, ASA and leptomeningeal anastomosis was limited and failed to provide sufficient blood flow and perfusion. We speculate that serious hypoperfusion without adequate collateral circulation was the main mechanism for medical therapy failure and recurrent ischemic events in these patients. The mechanism of artery-to-artery embolism based on hypoperfusion may also contribute to recurrent and progressive stroke in these patients (19). The ICVA occlusions caused hypoperfusion, and the resulting sluggish blood flow promoted the formation of clots that formed emboli. Obvious clots occurred in 41.9% (13/31) of these patients at and around the ICVA occlusion lesions in this study. Hypoperfusion and related changes in the dynamics of blood flow in the cerebral arteries also prevented the clearing of distal emboli.

The management of these patients is a medical dilemma. Patients who have hemodynamic compromise and comparatively slow infarct growth are highly likely to benefit from delayed recanalization. We demonstrated that endovascular recanalization appeared to be feasible for symptomatic atherosclerotic non-acute ICVA total occlusion attributable to hypoperfusion in this study. Based on our single-arm study results, we are not able to draw conclusions about the efficacy of delayed endovascular recanalization for these patients. However, all these patients presented with recurrent and progressive stroke despite aggressive medical therapy before the procedure, and it is encouraging to see that the hypoperfusion related lifestyle-limiting symptoms improved quickly within several days after the procedure in patients with successful recanalization. Over a median clinical follow-up duration of 11.0 months for the patients with successful recanalization, 74.1% (20/27) of these patients achieved a favorable clinical outcome (mRS score ≤2), and 88.9% (24/27) of these patients achieved an acceptable clinical outcome (mRS score ≤3). Except for one patient suffering symptomatic reocclusion 7.5 months after the procedure, there was no recurrent stroke during the clinical follow-up period in other patients with successful recanalization.

Although the symptomatic complication rate in this study was not very high (9.7%, 3/31), it should be emphasized that endovascular recanalization for non-acute ICVA occlusion is a high-risk procedure. Together failed procedure and symptomatic complication was 22.5% of patients, and all complications and failed procedures occurred in 32.3% of patients. Symptomatic atherosclerotic non-acute ICVA occlusion features a long lesion length (44.4%,12/27) and high clot burden (41.9%, 13/31), which makes the endovascular recanalization procedure more challenging in these patients than in patients who with non-acute middle cerebral artery occlusion or non-acute basilar artery (BA) occlusion (13, 14). The management of clots based on ICVA occlusion lesions was critical during the procedure. Transcatheter aspiration prior to angioplasty can effectively reduce the clot burden proximal to the occlusion segment, and this is a potential treatment to obtain better reperfusion and improve the prognosis. Angiographic follow-up demonstrated that continuous clot dissolution was achieved after successful recanalization in these patients. Embolization risk is very high and difficult to prevent in patients with clots distal to the occlusion segments. The clots breaking up and embolizing the distal segment of BA can cause life threatening complications. HRMRI is helpful in the diagnosis of ICVA occlusion and luminal thrombosis (20) and can help us to identify the subset of patients with high embolism risk before the procedure. At present, there is no embolic protection device to reduce embolization complications. The intervention procedure must be cautiously performed by experienced interventionalists, and emergency MT is critical for patients suffering embolization complications. The selection of eligible patients and the correct treatment for complications are equally critical.

There are several limitations of our study. First, this study is a single-center study; thus, selection bias may be possible. Other main limitations are its retrospective nature and a lack of a control arm. Prospective randomized controlled trials are needed to investigate whether endovascular recanalization compares favorably with aggressive medical management in these patients.

## Conclusions

Patients with symptomatic atherosclerotic non-acute ICVA total occlusion presented with recurrent and progressive ischemic events despite aggressive medical therapy. These patients had asymmetric VA due to unilateral hypoplasia, and ICVA total occlusions occurred in the dominant VA. The dominant ICVA total occlusion was characterized by long lesion length and high clot burden. These patients had relatively small multiple infarctions and large perfusion defects in the posterior circulation. Hypoperfusion without adequate circulation played a critical role in the recurrent ischemic events and lifestyle-limiting symptoms in these patients and endovascular recanalization appeared to be a technically feasible treatment for these patients.

## Data Availability Statement

The original contributions presented in the study are included in the article/supplementary materials, further inquiries can be directed to the corresponding author.

## Ethics Statement

The studies involving human participants were reviewed and approved by Institutional Review Board of the First Affiliated Hospital of Shandong First Medical University. The patients/participants provided their written informed consent to participate in this study.

## Author Contributions

WZ contributed to the design of the study, acquisition, analysis and interpretation of the data, drafting of the article and revision of the content, and final approval of the paper. JuZ, YM, and YZ contributed to the analysis of the data and the revision of content and the final approval of the article. JiZ, YS, LS, and MZ contributed to the analysis of the data and the final approval of the article. WW and HY contributed to the statistical analysis and the final approval of the article. JH contributed to the conception and design of the study, acquisition and analysis of the data, drafting of the article, revision of the content, and final approval of the article. All authors contributed to the article and approved the submitted version.

## Conflict of Interest

The authors declare that the research was conducted in the absence of any commercial or financial relationships that could be construed as a potential conflict of interest.
