# Supplementary material for: Symptomatic Atherosclerotic Non-acute Intracranial Vertebral Artery Total Occlusion: Clinical Features, Imaging Characteristics, Endovascular Recanalization, and Follow-up Outcomes
Source: Front Neurol. 2020 Nov 16;11:598795. doi: 10.3389/fneur.2020.598795 (PMC7703109; doi:10.3389/fneur.2020.598795)
Supplement: Supplementary file 1 [file Table_1.docx]

**Supplemental table 1** Clinical summary of 31 patients undergoing endovascular recanalization

| Patient Number | NIHSS  Score Pre-procedure | mRS score Pre-procedure | Pc- ASPECTS  on DWI | Time  between  initial  symptoms  and  treatment  (days) | Time  between  image documented  occlusion and  treatment  (days) | Successful recanaliztion (yes/not) | Post-procedure perfusion (TICI grade) | Complications | 30- day mRS score | the last follow up mRS score | Recurrent TIAs or  strokes at the last follow up |
| --- | --- | --- | --- | --- | --- | --- | --- | --- | --- | --- | --- |
| 1 | 9 | 4 | 6 | 22 | 15 | yes | 3 | no | 3 | 2 | no |
| 2 | 4 | 2 | 5 | 64 | 40 | yes | 3 | perforating branch occlusion | 3 | 1 | no |
| 3 | 3 | 3 | 6 | 30 | 30 | yes | 3 | intracranial hemorrhage | 1 | 1 | no |
| 4 | 0 | 2 | 7 | 96 | 22 | yes | 3 | no | 0 | 0 | no |
| 5 | 7 | 3 | 1 | 6 | 4 | yes | 3 | no | 1 | 1 | no |
| 6 | 3 | 2 | 7 | 180 | 180 | not | 0 | no | 2 | NA (dead because of myocardial infarction) | recurrent TIAs |
| 7 | 7 | 3 | 2 | 90 | 90 | yes | 3 | no | NA (dead because of multiple organ failure) | NA | NA |
| 8 | 16 | 4 | 1 | 11 | 9 | yes | 2b | no | 4 | 4 | no |
| 9 | 0 | 2 | 5 | 12 | 12 | yes | 3 | no | 1 | 0 | no |
| 10 | 3 | 2 | 6 | 52 | 5 | yes | 3 | no | 1 | 1 | no |
| 11 | 5 | 2 | 6 | 65 | 65 | not | 0 | no | 2 | 2 | no |
| 12 | 4 | 3 | 7 | 14 | 7 | yes | 3 | no | 2 | 1 | no |
| 13 | 16 | 5 | 2 | 16 | 6 | yes | 3 | no | 4 | 4 | no |
| 14 | 2 | 4 | 7 | 15 | 8 | yes | 3 | no | 1 | 0 | no |
| 15 | 7 | 4 | 4 | 34 | 6 | yes | 3 | embolization | 1 | 1 | no |
| 16 | 10 | 4 | 6 | 33 | 29 | yes | 3 | no | 3 | 3 | no |
| 17 | 1 | 2 | 8 | 33 | 12 | yes | 3 | no | 0 | 0 | one recurrent stroke |
| 18 | 0 | 2 | 8 | 35 | 9 | yes | 3 | no | 0 | 0 | no |
| 19 | 3 | 3 | 6 | 16 | 8 | yes | 3 | no | 1 | 0 | no |
| 20 | 18 | 4 | 5 | 18 | 14 | yes | 3 | no | 4 | 3 | no |
| 21 | 6 | 3 | 1 | 32 | 7 | yes | 3 | no | 1 | 0 | no |
| 22 | 8 | 4 | 5 | 13 | 6 | yes | 3 | no | 0 | 0 | no |
| 23 | 6 | 4 | 5 | 10 | 5 | yes | 3 | perforating branch occlusion | 3 | 3 | no |
| 24 | 2 | 2 | 7 | 23 | 18 | not | 0 | dissection | 3 | 3 | no |
| 25 | 8 | 4 | 7 | 11 | 6 | yes | 2b | no | 3 | 3 | no |
| 26 | 0 | 2 | 6 | 55 | 44 | yes | 3 | no | 0 | 0 | no |
| 27 | 5 | 2 | 6 | 17 | 15 | yes | 3 | no | 0 | 0 | no |
| 28 | 0 | 2 | 6 | 22 | 22 | not | 0 | no | 2 | 2 | recurrent TIAs |
| 29 | 2 | 2 | 6 | 120 | 58 | yes | 3 | no | 1 | 0 | no |
| 30 | 4 | 3 | 7 | 15 | 14 | yes | 3 | embolization | 1 | 1 | no |
| 31 | 4 | 4 | 6 | 105 | 104 | yes | 3 | no | 2 | 2 | no |

NIHSS: National Institutes of Health Stroke Scale; mRS: modified Rankin Scale; pc-ASPECTS: posterior circulation acute stroke prognosis early CT score;

DWI: diffusion-weighted imaging; TICI, thrombolysis in cerebral infarction; TIAs: transient ischemic attacks; NA: not applicable.
